# 

**Published:** 1936

**Authors:** 


### CONTENTS.

| | *Page* |
|---|---|
| In Memoriam: King George V. | 1 |
| Photography and Medicine. By A. E. Iles, O.B.E., M.B., F.R.C.S. | 3 |
| Ultra-Microscopic Examination of the Blood-Serum in Disease. By B. A. Peters, M.D., D.P.H. | 17 |
| Bristol Eye Hospital. By The Editor | 35 |
| The Position of the Voluntary Hospitals in the National Health Services. By Robert F. Barclay, LL.D. | 39 |
| Reviews of Books | 45 |
| Editorial Notes | 54 |
| Obituary. Emeritus-Professor Conwy Lloyd Morgan, F.R.S., LL.D. | 59 |
| Meetings of Societies | 61 |
| The Medical Library of the University of Bristol | 62 |
| Publications Received | 65 |
| Local Medical Notes | 66 |

